# The utility of bioenergetics modelling in quantifying predation rates of marine apex predators: Ecological and fisheries implications

**DOI:** 10.1038/s41598-017-13388-y

**Published:** 2017-10-11

**Authors:** A. Barnett, M. Braccini, C. L. Dudgeon, N. L. Payne, K. G. Abrantes, M. Sheaves, E. P. Snelling

**Affiliations:** 1 College of Marine and Environmental Sciences, James Cook University, Townsville, Queensland 4811 Australia; 2 Western Australian Fisheries and Marine Research Laboratories, North Beach, Western Australia 6920 Australia; 3 School of Biomedical Sciences, University of Queensland, St. Lucia, Queensland 4072 Australia; 4 University of Roehampton, Holybourne Avenue, London, SW15 4JD United Kingdom; 5 Brain Function Research Group, School of Physiology, University of the Witwatersrand, Johannesburg, Gauteng, 2193 South Africa

## Abstract

Predators play a crucial role in the structure and function of ecosystems. However, the magnitude of this role is often unclear, particularly for large marine predators, as predation rates are difficult to measure directly. If relevant biotic and abiotic parameters can be obtained, then bioenergetics modelling offers an alternative approach to estimating predation rates, and can provide new insights into ecological processes. We integrate demographic and ecological data for a marine apex predator, the broadnose sevengill shark *Notorynchus cepedianus*, with energetics data from the literature, to construct a bioenergetics model to quantify predation rates on key fisheries species in Norfolk Bay, Australia. We account for the uncertainty in model parameters by incorporating parameter confidence through Monte Carlo simulations and running alternative variants of the model. Model and parameter variants provide alternative estimates of predation rates. Our simplest model estimates that ca. 1130 ± 137 *N*. *cepedianus* individuals consume 11,379 (95% CI: 11,111–11,648) gummy sharks *Mustelus antarcticus* (~21 tonnes) over a 36-week period in Norfolk Bay, which represents a considerable contribution to total predation mortality on this key fishery species. This study demonstrates how the integration of ecology and fisheries science can provide information for ecosystem and fisheries management.

## Introduction

It is well-accepted that predators play crucial roles in the structure and function of ecosystems, but quantifying rates of predation remains difficult^1–4^. Predation pressure is often inferred^5–7^, and a number of studies have quantified non-consumptive effects (risk effects) on prey^8^. Quantifying predation rates provides information for better detecting ecological processes, defining predators’ roles in different systems, and determining the strength of species interactions^1,9^. It can also assist applications such as providing more precise data for ecosystem models, ensuring sustainable harvests of prey species, and improving estimates of natural mortality in commercially fished populations^10–12^. In fisheries research and management, natural mortality is a difficult parameter to quantify, yet often one of the most important^10,13^. For example, mortality from predation can exceed that from fisheries, and so estimates of mortality from predation can add important information to stock assessment models^10,14^.

Determining the direct effects of predation requires information that is often difficult to obtain, including the rate of prey consumption, which is dictated by the predator’s metabolic rate and influenced by the energetic value of its prey^1,15^. Another obstacle is obtaining reliable estimates of absolute abundance of predators (as opposed to relative abundance indices), so that individual prey consumption rates can be scaled-up to the population level^15^. For example, several studies use different methods to assess the rates of prey consumption for killer whales *Orcinus orca*, but none of these studies quantify *O*. *orca* population sizes (see Noren^15^ and references therein). Studies that estimate marine predator abundance for use with bioenergetics models not only quantify predation at the population level, but also contribute significantly to our understanding of the role of marine predators in the structure and function of ecosystems^1,16–19^. For example, incorporating abundance estimates into a bioenergetics model for adult grey reef sharks *Carcharhinus amblyrhynchos* demonstrates the significant contribution of fish spawning aggregations to meeting the energetic demands of a population in Fakarava Pass, French Polynesia^19^.

Given the increasing acceptance of ecosystem and multi-species approaches to fisheries management, integrating ecological methods, such as identifying and quantifying key species interactions, is believed to be a primary direction for fisheries science^12,20^. However, the general uncertainty of species interactions and the poor understanding of the ecology of many species (e.g. diet, population dynamics, spatial distribution, habitat use, etc.) can hinder multi-species approaches^21^.

**Figure 1:**
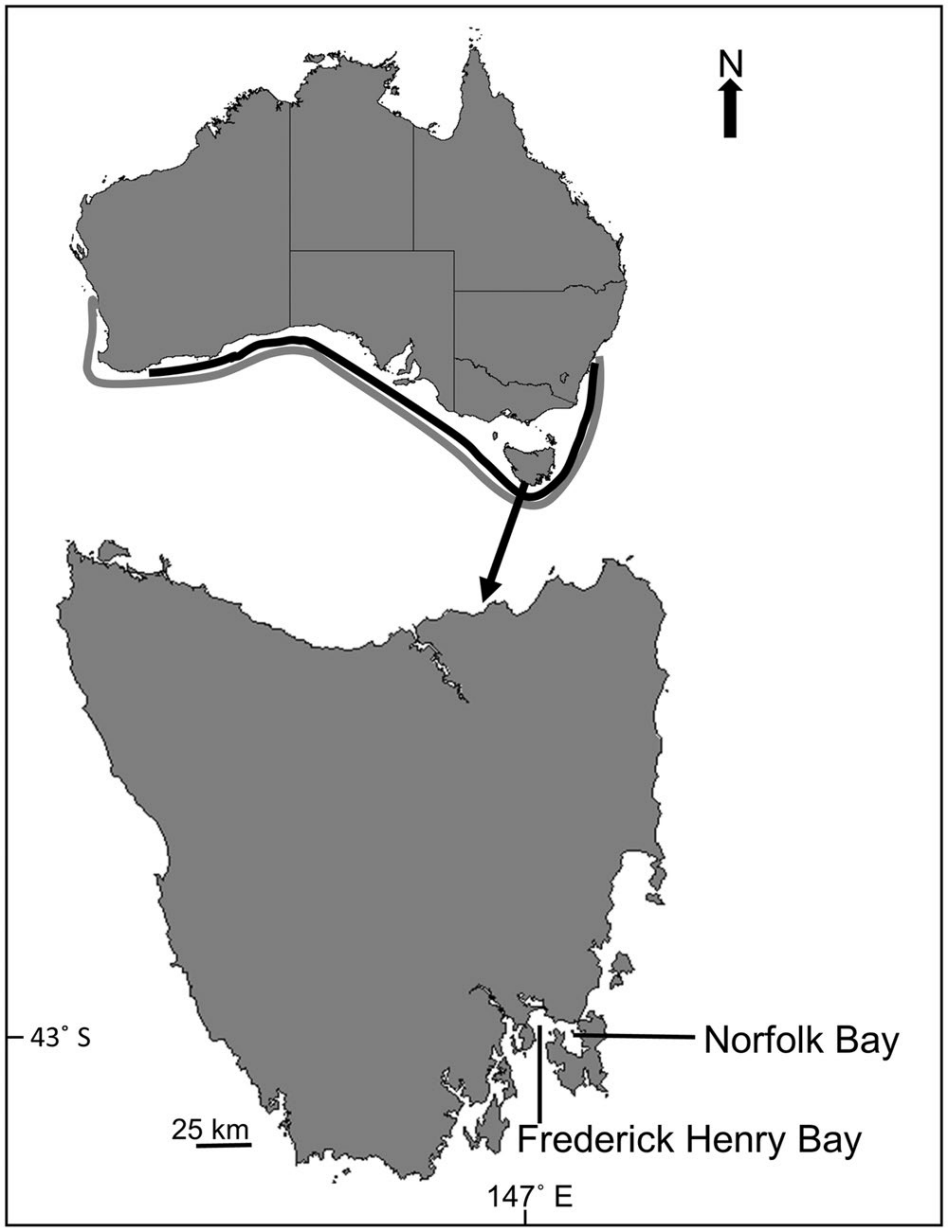
Map showing study area Norfolk Bay in southern Tasmania, Australia. Grey and black lines represents gummy shark *Mustelus antarcticus* (grey) and sevengill shark *Notorynchus cepedianus* (black) distributions in southern Australia (approximately from line into coast). Figure generated in Powerpoint (Microsoft Office 2013).

A suite of relevant studies on the broadnose sevengill shark *Notorynchus cepedianus* in Norfolk Bay, Tasmania, southern Australia (Fig. 1), provides a rare opportunity to estimate predation rates by a seasonal population of a marine apex predator. This shark is a fishery-associated species with a broad global distribution^22^. It is a common bycatch species (with a low commercial value) in the southern shark fishery of Australia, and is the most significant predator of commercially important juvenile shark species^23–25^. *Notorynchus cepedianus* occurs in high numbers in coastal Tasmania over the warmer months of the year (spring to autumn), where it exerts significant predation pressure on prey inhabiting those waters^6,24–26^, and likely plays a crucial role as one of the key apex predators in temperate waters^6^. This study integrates a suite of available information on the demography, activity, and diet of *N*. *cepedianus* with information on energetics from the literature into a bioenergetics model. Uncertainty in model input parameters is accounted for by incorporating parameter confidence through Monte Carlo simulations and running alternative variants of the model. The model variants are used (1) to quantify the overall role of *N*. *cepedianus* as the apex predator in Norfolk Bay and (2) to specifically estimate predation mortality of a key fisheries species, the gummy shark *Mustelus antarcticus*, in an elasmobranch protected area. Model and parameter variants provide alternative estimates of predation rates for all prey species. The strengths and weaknesses of model variants and parameter uncertainty are discussed.

## Results

A bioenergetics model was constructed to estimate predation rates by *N*. *cepedianus* on the gummy shark *Mustelus antarcticus* and on other key prey species, in Norfolk Bay, Tasmania (Fig. 1). The model was applied over a 36 week period from September to May (spring-summer-autumn seasons) during which *N*. *cepedianus* are known to aggregate in the bay. The model incorporates (1) routine energy expenditure of free-swimming *N*. *cepedianus* in Norfolk Bay, (2) population size of *N*. *cepedianus* in Norfolk Bay, (3) demographic information for *N*. *cepedianus* and for prey species, (4) energy content of various prey species inhabiting Norfolk Bay, and the (5) relative importance of these prey species to the diet of *N*. *cepedianus* in Norfolk Bay. To account for uncertainty in model structure and input parameters, we built variants of the model based on three alternative techniques for measuring diet composition (see Methods). Separate models were run for each of the three diet variants taking into account two estimates of *N*. *cepedianus* population sizes in Norfolk Bay during the 36 week period: 562 ± 71 and 1130 ± 137 sharks. For each of the six scenarios considered, 1000 Monte Carlo simulations were performed to account for uncertainty in model parameters (see Methods).

Our sampling data indicate that *N*. *cepedianus* occurring in Norfolk Bay have an average body mass of 42 kg (Table 1). Accordingly, we estimate that the average free-swimming routine energy expenditure of *N*. *cepedianus* in Norfolk Bay is approximately 1150 kJ day^−1^ at a water temperature of 16.7 °C (assuming Q_10_ = 2.2 and RQ = 0.88). During spring, when water temperature drops to 14.0 °C, routine energy expenditure is predicted to decrease to 930 kJ day^−1^, and during summer, when water temperature increases to 19.1 °C, routine energy expenditure is predicted to increase to 1390 kJ day^−1^ (Q_10_ = 2.2 and RQ = 0.88).

**Table 1:** Parameter used in the bioenergetics model.

Species (or group)	TL or DW (cm) (mean ± SD; range)	M_b_ (kg) (mean ± SD; range)	TL or DW to M_b_relationship	Reference for TL or DW to M_b_ conversion	Tissue energy-density (kcal/g)	Reference for tissue energy-density
Sevengill shark *Notorynchus cepedianus*	208 ± 35; 105–270	42.0 ± 22.0; 2.8–88.0	Females: M_b_ = 0.003TL^2^–0.42TL + 19.501 (R² = 0.996; *n* = 216) Males: M_b_ = 0.002TL^2^–0.22TL + 8.803 (R² = 0.98, *n* = 78)	42		
Fur seal (FS) *Arctocephalus pusillus;* Other mammals (M) (undigested contents only)		2.1 ± 1.3; 0.7–4.0		Barnett unpub. data	2.5 (FS) 2.4 (M)	FS based on fur seal species; M based on the average of pinniped & whale estimates^1,18,58^
Gummy shark *Mustelus antarcticus*	74 ± 20; 28–143	1.8 ± 1.6; 0.1–11.5	Females: M_b_ = 0.93 × 10^−29^ × 1.07 × (TL × 10)^3.21^ (R^2^ = 0.95; *n* = 1077) Males: M_b_ = 4.210.10^−9^ × 1.016 × (TL × 10)^2.976^ (R^2^ = 0.93; *n* = 862)	59	1.5	Based on *Squalus acanthias* ^60^
School shark *Galeorhinus galeus*	66 ± 16; 31–113	1.2 ± 0.8; 0.1–5.2	Same as gummy shark	59	1.5	60
Dogshark *Squalus acanthias*	54 ± 11; 19–94	0.73 ± 0.5; 0.03–4.2	M_b_ = 0.05TL^2.6^ × 1000 (R^2^ = 0.96*; n* = 32)	61	1.5	60
Eagle rays *Myliobatis tenuicaudatus*.	81 ± 14; 70–110	9.1 ± 5.8; 0.9–48.6	M_b_ = 2.76 × 10^−05^ × DW^2.9^ (R^2^ = 0.95; *n* = 393)	62	1.1	Based on batoid species in (60, 16)
Melbourne skate *Spiniraja whitleyi*	87 ± 29; 33–196	18.2 ± 17.7; 0.1–124.6	M_b_ = 0.005DW^2^–0.29DW + 4.65 (R^2^ = 0.96; *n* = 72)	Treloar unpub. data	1.1	60
Banded stingaree *Urolophus cruciatus*	18 ± 4; 9–30	0.3 ± 0.2; 0.03–1.1	M_b_ = 0.002DW^2^–0.03DW + 0.14 (R² = 0.96; *n* = 75)	Yick unpub. data	1.1	60
Elephantfish *Callorhynchus milii*	73 ± 9; 45–100	2.5 ± 1.2; 0.4–7.3	Females: M_b_ = 7.54e^−10^ × (TL × 10)^3.3^ Males: M_b_ = 6.3e^−11^ × (TL × 10)^3.7^	Braccini unpub. data	1.0	Based on *Callorhynchus callorhynchus* ^60^
Teleosts		0.8		Estimated average weight for multiple species combined	1.5	Average of all teleosts in^60^
Cephalopods (mainly arrow squid)		0.7		Estimated weight of squid^63,64^	1.5	60

Because entire seals/mammals were not consumed by an individual *N*. *cepedianus*, Only weight of undigested mammal occurring in stomach samples was used to obtain average weight of mammal consumed. TL = total length, DW = disc width for batoids, M_b_ = body mass.

**Figure 2:**
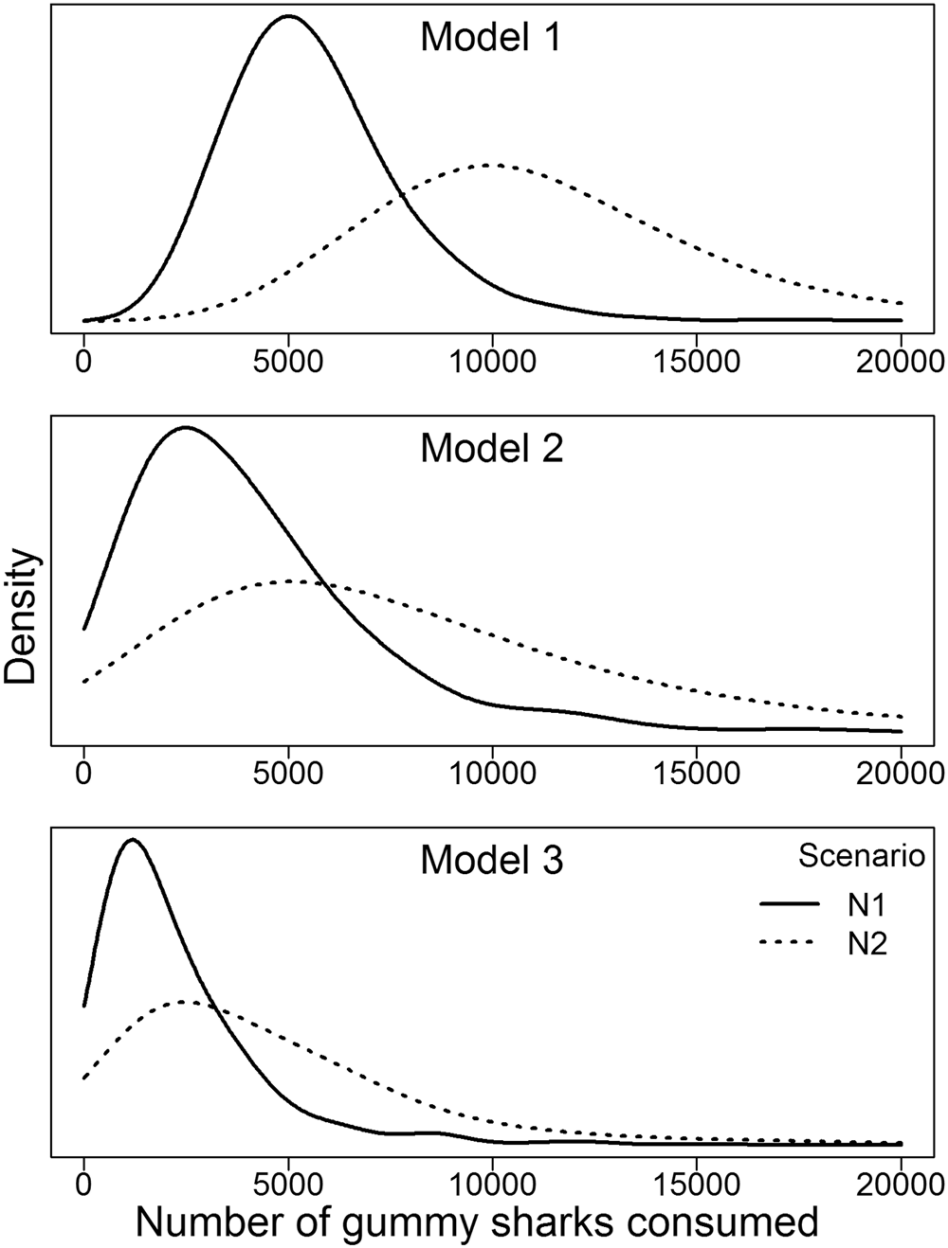
Probability of *N*. *cepedianus* predation on gummy shark *M*. *antarcticus* based on the outputs of the three variants of the bioenergetics model, fitted by a log normal distribution.

**Figure 3:**
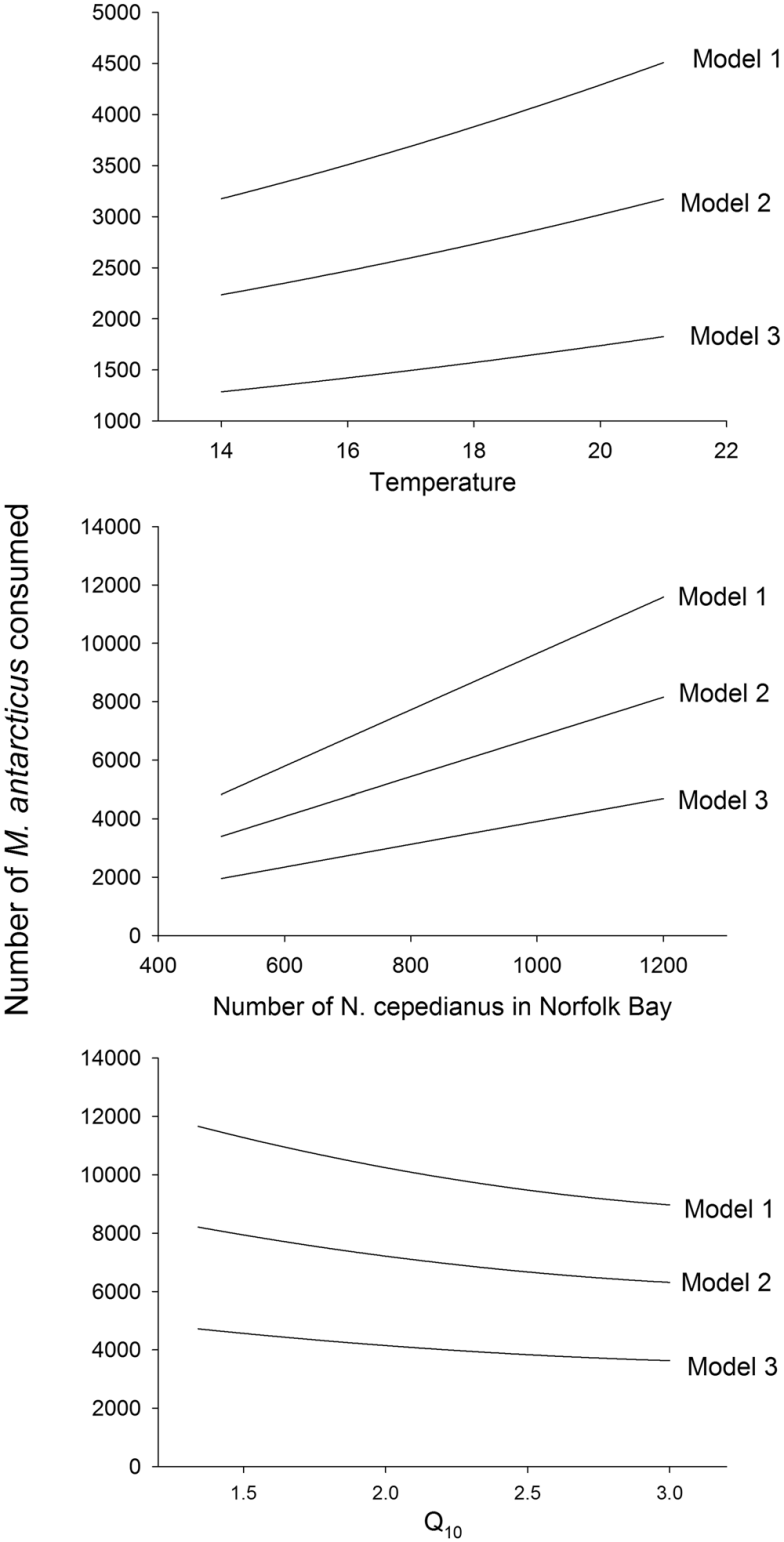
Variation in the predicted *N*. *cepedianus* predation on gummy shark *M*. *antarcticus* for the three model variants, and the effect of temperature, Q_10_ and population size on the model outputs. Temperature and Q_10_ are based on the population estimates of 1130 *N*. *cepedianus* occurring in Norfolk Bay.

The number of prey consumed over the 36 week period, along with the relative importance of the different prey species (or groups), varied among the three models and six scenarios (Table 2, Fig. 2). For example, the consumption rate of *M*. *antarcticus* differed among models (Fig. 2, Table 2). Moreover, uncertainties and variations in model parameters that likely fluctuate within and between years (i.e. water temperature and number of *N*. *cepedianus*) or parameters lacking specific data for *N*. *cepedianus* (Q_10_) produced differences in modelled prey consumption rates (Fig. 3). Changes in all three parameters led to differences in modelled consumption rates of *M*. *antarcticus* (Fig. 3).

**Table 2:** Estimated number (with 95% confidence interval range) of each prey type consumed by *N*. *cepedianus* over the 36-week sampling year in Norfolk Bay based on three model variants and two scenarios of *N*. *cepedianus* population size^26^.

Species (or groups)	M1 N1	M1 N2	M2 N1	M2 N2	M3 N1	M3 N2
Fur seal *Arctocephalus pusillus*			49 (47–50)	98 (95–102)	10 (10–11)	20 (19–21)
Other mammal			15 (14–15)	29 (28–31)	6 (6–7)	13 (12–13)
Gummy shark *Mustelus antarcticus*	5656 (5523–5789)	11379 (11111–11648)	4085 (3897–4273)	8294 (7913–8675)	2241 (2119–2364)	4653 (4396–4911)
School shark *Galeorhinus galeus*			499 (473–524)	1009 (958–1061)	131 (123–139)	266 (252–281)
Dogshark *Squalus acanthias*			1061 (1005–1118)	2179 (2052–2305)	815 (770–860)	1616 (1527–1705)
Unidentified shark			4657 (4403–4910)	9357 (8842–9872)	946 (880–1012)	1991 (1859–2123)
Eagle rays *Myliobatis tenuicaudatus*			495 (471–520)	1006 (957–1056)	882 (843–922)	1862 (1778–1947)
Melbourne skate *Spiniraja whitleyi*			317 (299–334)	654 (617–690)	1133 (1086–1179)	2297 (2206–2387)
Banded stingaree *Urolophus cruciatus*			3359 (3171–3548)	6775 (6407–7143)	744 (701–787)	1406 (1328–1484)
Unidentified batoid			88 (80–97)	177 (160–193)	611 (569–652)	1195 (1101–1289)
Elephantfish *Callorhynchus milii*			195 (187–204)	396 (378–414)	308 (294–321)	610 (583–636)
Teleosts			2404 (2335–2473)	4858 (4718–4998)	3429 (3161–3698)	6554 (6041–7066)
Cephalopods (mainly arrow squid)			136 (132–140)	275 (267–283)	536 (518–554)	1083 (1048–1118)

M1–3 = model variant 1–3, N1 = population of 562, and N2 = population of 1130.

### Model variant 1

Assuming that 25% of *N*. *cepedianus* population in Norfolk Bay contains *M*. *antarcticus* at any given time^25^, and that *N*. *cepedianus* abundance in the bay varies between 562 and 1130 individuals throughout spring to autumn^22^, this model estimates that between 5656 (95% CI: 5523–5789) and 11,379 (95% CI: 11111–11648) *M*. *antarcticus* are consumed over the 36 week period (Fig. 2, Table 2).

### Model variant 2

The high relative weight of mammal in the diet^25^ influences this model’s output, with up to 127 mammals (98 fur seals) consumed over the 36-week period (Table 2). This model predicts that between 4085 (95% CI: 3897–4273) and 8294 (95% CI: 7913–8675) *M*. *antarcticus* are consumed over the 36 week period annually in Norfolk Bay (Table 2).

### Model variant 3

The influence of marine mammal is reduced considerably in this model compared to model variant 2, with a maximum of 33 mammals (20 fur seals) consumed over the 36-week period (Table 2). Batoids, most notably *Myliobatis tenuicaudatus* and *Spiniraja whitleyi*, have a stronger influence on this model’s output (Table 2). This is influenced by the large average weight of skates and eagle rays (Table 1), resulting in those species having the highest average relative weight, i.e. skates 43% and eagle rays 16% (Appendix S1). All shark species (or groups) were consumed in lower numbers compared to model variant 2, including *M*. *antarcticus* (Table 2). Between 2241 (95% CI: 2119–2364) and 4653 (95% CI: 4396–4911) *M*. *antarcticus* individuals were estimated to be consumed over the 36-week period annually in Norfolk Bay. Estimates of teleost and cephalopod consumption were also higher in model variant 3 than in model variant 2 (Table 2).

## Discussion

### Bioenergetics model

Quantifying predation rates by marine predator populations is a significant challenge in ecology and fisheries science. Previous studies that have incorporated predator abundance estimates with bioenergetics models to quantify predation mainly includes marine mammals^17,18,27^, and fisheries species, such as tunas^28,29^. Although previous studies have constructed bioenergetics models for sharks (e.g.^30–32^), few have incorporated abundance estimates that allow consumption rates to be scaled up to the population level^16,19^. The paucity of marine studies is not surprising given the difficulties associated with observing predators in general, and obtaining the suite of relevant parameters required for bioenergetics models. To compensate for this, these models are often based on best available assumptions, such as published data from similar species. Here, we present three model simulations using a combination of field data for predator and prey demographics and ecology, and information on energetics from the literature. Model 1 centres on the main strengths of available data, which is a very good understanding of *N*. *cepedianus* and *M*. *antarcticus* demographics and ecology in Norfolk Bay. This model provides estimates of predation mortality for *M*. *antarcticus*, a key fisheries species, in an area closed to commercial and recreational fishing to protect the population (see below). The second and third models take a broader system approach, and were populated with as much information that could be obtained for other prey species.

For fisheries applications, model 1 is the simplest to obtain data for as it only requires data for the predator and the target fisheries species. However, model 1 does not consider the input of other prey species (number or energetic value) to the diets of *N*. *cepedianus*, and this could be driving the higher estimates of *M*. *antarcticus* consumed compared to the other models. Still, higher estimates of *M*. *antarcticus* consumption may be the most appropriate if a conservative approach is considered best for fisheries management^10^. For ecosystem studies, model 3 is likely more accurate than model 2. Model 2 (based on % weight) overestimates mammal consumption. Marine mammals are not seen in Norfolk Bay in large numbers (Barnett pers. obs.), and the closest haul-out site is over 50 km away^25^. Either predation is significantly less, as predicted in model 3, or *N*. *cepedianus* are feeding on mammals elsewhere before entering Norfolk Bay. In general, % weight alone is not a good indicator of prey value as issues such as partial and differential digestion can provide ambiguous interpretations^33^. Furthermore, as digestion proceeds, only the components that are indigestible, or slow to digest, remain identifiable and potentially measureable^33^. For example, cephalopod beaks and fish otoliths are often all that remains of these animals in stomach samples. In model 3, the increase in the importance of batoids is primarily driven by the large average size of *S*. *whitleyi* and *M*. *tenuicaudatus* caught in Norfolk Bay, which influences their average % weight in the model (Appendix S1). The importance of batoids is likely overestimated in this model because smaller size classes of skates and eagle rays were not caught in the fishing gears used (inflating average size of batoids), and the likelihood that larger batoids are consumed by multiple *N*. *cepedianus*. This had some effect on the model output, such as lowering the estimates of *M*. *antarcticus* consumed (Table 2).

A major challenge with bioenergetics models is the level of uncertainty associated with input parameter values^27^. A few of the parameters in our model undoubtedly introduce some uncertainty. For example, Q_10_ for *N*. *cepedianus* is unknown, and available literature suggests it could be between 1.3 and 3.0. This difference in Q_10_ can lead to estimates that differ by ~2000 individual *M*. *antarcticus* consumed over the sampling year (Fig. 3).There could be some uncertainty regarding dietary composition, as stomach content analysis only provides information for a snapshot in time. However, stomach data were collected over three years and studies show that *N*. *cepedianus* diet composition can be linked to prey abundance^25^, as discussed in fisheries section below. Thus, uncertainty associated with parameters such as Q_10_, water temperature, *N*. *cepedianus* body mass (which are all related to uncertainty in metabolic rate), other metabolic rate parameters (power equation coefficient and power equation exponent) and prey composition, were factored into our analysis by running Monte Carlo simulations. Furthermore, uncertainty in *N*. *cepedianus* abundance was also factored in by using two different scenarios for each of the three versions of the model. Models can be updated when improved estimates for the different parameters become available. For instance, when technology becomes available for sharks of this size, conducting respirometry experiments and integrating field-derived activity data for *N*. *cepedianus* to determine species-specific metabolic rate may improve estimates of energetics and prey consumption^34^, as it addresses the most likely variable component of our bioenergetics model. Considering the best available information, we assumed similar activity for day and night based on *N*. *cepedianus* cruising speeds calculated from acoustic telemetry not being significantly different^35^. However, given that *N*. *cepedianus* appear to move over a larger area at night and the significant increase in movement rates between cruising and burst speeds^35^, field activity studies may also elucidate diel patterns in active metabolic rates.

### Fisheries implications

A number of coastal areas in southern Australia prohibit the taking of any elasmobranchs to protect neonate and juvenile *M*. *antarcticus* ^23^. Based on the average weight of *M*. *antarcticus* in Norfolk Bay (1.8 ± 1.6 kg), the consumption of 2241 to 11,379 individuals by *N*. *cepedianus* equates to an annual consumption of between 4 and 21 tonnes. This is the first estimate of the predation component of natural mortality for *M*. *antarcticus*. Norfolk Bay is a relatively small area (~180 km^2^) within the spatial distribution of *M*. *antarcticus* and *N*. *cepedianus* (Fig. 1) so, at the broader population level (distribution in Australian temperate waters), the total annual consumption of *M*. *antarcticus* by *N*. *cepedianus* could be at the same order of magnitude of the current catch quota for *M*. *antarcticus*, in the Southern and Eastern Scalefish and Shark Fishery (1836 tonnes)^36^.

It is however important to recognise that predation rates on each species by *N*. *cepedianus* likely varies between locations, depending on differences in prey availability, water temperature and *N*. *cepedianus* abundance. For example, the high consumption of *M*. *antarcticus* coincides with it being one of the most relative abundant prey in Norfolk Bay and neighbouring bays^6,25^ (Appendix S2). Similarly, increases in consumption of *S*. *acanthias* that coincide with high relative abundance in the neighbouring Derwent Estuary have been reported^25^. Likewise, in Tasmania and southern Africa, *N*. *cepedianus* consumed more marine mammals in the region with the highest concentration of seal rookeries, while chondrichthyans were the most important prey in the other regions^25,37,38^. Catches of some other species are high in neighbouring bays compared to Norfolk Bay. For example, *S*. *acanthias* and *C*. *milii* are caught in higher numbers in the neighbouring Fredrick Henry Bay and Pittwater, respectively. The greater presence of these species would likely result in an increase in their occurrence in the diet of *N*. *cepedianus* at those locations (Appendix S2). In general, *N*. *cepedianus* target other elasmobranchs and marine mammals globally, but the main species consumed within these groups can vary^25^. However, sharks from the genus *Mustelus* (family Triakidae) and other triakid species are the most common prey consumed by *N*. *cepedianus* in all regions globally^25^, suggesting that, when they are abundant, triakids are the main prey.

Besides the aforementioned links to fisheries, *N*. *cepedianus* is also linked to fisheries by being an important predator of elasmobranchs and pinnipeds that compete with fisheries^39,40^. In particular, fur seal *Arctocephalus pusillus* numbers have recovered significantly in Australia since their protection in 1975 and many in the fishing industry deem them as competitors for diminishing resources^40^. In areas of southern Australia with greater pinniped abundance, *N*. *cepedianus* likely consume more pinnipeds, and probably play a role in reducing pinniped competition with fisheries.

### Role of Notorynchus cepedianus

Previous work has inferred high predation pressure by *N*. *cepedianus* in coastal areas, as reviewed by Barnett and colleagues^22^. The current study shows that in areas of high abundance, *N*. *cepedianus* have significant impacts on the various prey species and very likely play an important role in ecosystem dynamics, e.g. top-down control of ecosystems. *Notorynchus cepedianus* consume the same prey as white sharks *Carcharodon carcharias*, including marine mammals, teleosts and elasmobranchs^22,37,41^. However, despite rivalling *C*. *carcharias* as the dominant apex predator in temperate waters, the ecosystem importance of *N*. *cepedianus* has been largely overlooked. Indeed, *N*. *cepedianus* arguably have a greater influence on top-down effects, such as ecosystem structure and controlling mesopredator numbers, as available information suggests they are much more abundant across temperate systems than *C*. *carcharias* ^26,42^.

Given that water temperature plays an important role in predation rates (Fig. 3), increases in water temperature due to climate change could change the dynamics in shallow coastal bays such as Norfolk Bay. For example, model 1 predicts that 1130 *N*. *cepedianus* would consume ~1000 more *M*. *antarcticus* in summer compared to spring (Fig. 3). Tasmania is considered particularly susceptible to climate change, with warmer waters extending the southern range of some species along the east coast of Australia^43^. However, Tasmania is the most southern coastal area, and there is nowhere further south for *N*. *cepedianus* to move, and so if temperatures increase, they will need to adapt by increasing predation rates to meet the increasing energetic demands, or by spending more time in cooler deeper waters, which may affect their diet.

In conclusion, the integration of multiple types of information from a comprehensive suite of studies on *N*. *cepedianus* and its prey in Norfolk Bay has culminated in one of the first quantified estimates of predation for an apex predator shark species. *Notorynchus cepedianus* is an undervalued predator in coastal systems that competes directly with fisheries for common food resources. Given the wide distribution of *N*. *cepedianus*, they likely play an important role in ecosystem dynamics in temperate systems globally. Furthermore, *N*. *cepedianus* are intrinsically linked to fisheries, making them a good case study to show how the integration of ecology into fisheries science, i.e. “fisheries ecology”, can provide data that can be used for applied outcomes in ecosystem and fisheries management.

## Methods

### Study site

Norfolk Bay is a relatively shallow (maximum depth of ~20 m), semi-enclosed bay, covering an area of ~180 km^2^, off the southeast coast of Tasmania, Australia (Fig. 1). Norfolk Bay is located within a shark refuge area, and as such, commercial and recreational fishing for elasmobranchs is not permitted. The bay provides an important feeding site for the broadnose sevengill shark *Notorynchus cepedianus* and aggregations occur in the bay from September to May^26,44^. In this study, the energetics and predation habits of *N*. *cepedianus* in Norfolk Bay were analysed over this 36-week period, encompassing the spring-summer-autumn seasons. All field work was conducted under an Australian Fisheries Management Authority Scientific Permit (#901193) and the methods were approved by the University of Tasmania Animal Ethics Committee (#A0012578).

### Routine energy expenditure of *N*. *cepedianus*

The bioenergetics model constructed for this study estimates predation rates by *N*. *cepedianus* on the gummy shark *Mustelus antarcticus*, as well as other prey species, in Norfolk Bay from spring to autumn. To achieve this aim, an estimate of the routine energy expenditure of free-swimming *N*. *cepedianus* in Norfolk Bay was required. Over a period of two years and 3 months (to include 3 summers), 294 *N*. *cepedianus* individuals were caught in Norfolk Bay using longline fishing methods^24^. For each of these sharks, length measurements, sex and stomach contents (from stomach flushing) were recorded^45^. Since *N*. *cepedianus* is an ectotherm, the routine energy expenditure (*MR*; mg O_2_ h^−1^) was calculated for each of these 294 individuals using the allometric power equation for a group of free-swimming ectothermic sharks species, *MR* = 214*M*_b_^0.79^, correct to 20 °C^46^, where *M*_b_ is body mass in kg, which was estimated for each individual using sex-specific *N*. *cepedianus* length-weight data^47^ (Table 1). This estimate of overall mean routine energy expenditure is unlikely to vary across a 24-h cycle owing to activity measurements that indicate the rate of movement by *N*. *cepedianus* in Norfolk Bay is relatively constant during the day and night^35^. The routine energy expenditure of each individual *N*. *cepedianus* was, however, adjusted according to seasonal mean variation in water temperature (measured in the adjoining Fredrick Henry Bay: spring 14.0 °C, summer 19.1 °C, autumn 16.9 °C, overall mean 16.7 °C) using a uniform distribution Q_10_ between 1.3 and 3.0. This Q_10_ range was applied because it represents the temperature sensitivity of metabolism reported across nine species of elasmobranchs^48,49^. We also allocated an additional 5% energy expenditure to account for the cost of growth, which is estimated at approximately 8.7–14.6 cm year^−1^ given the size range of *N*. *cepedianus* in Norfolk Bay^50^, and is consistent with the little available literature that suggests between 3.5 and 7.2% of metabolic rate is invested in growth in sharks^30,51^. We also allocated another 5% increase in energy expenditure to account for the cost of reproduction, but only in one-third of the mature females, which was based on *N*. *cepedianus* probably having a three-year reproductive cycle^52^. Some mature females in Norfolk Bay have been found to be ovulating, in the initial stages of pregnancy, or starting a new vitellogenic cycle^52^. The cost of reproduction is unlikely to be much higher because Norfolk Bay and its neighbouring coastal areas are not used as pupping grounds, mating rarely occurs there, and most female *N*. *cepedianus* are non-gravid while in Norfolk Bay^52^.

After accounting for the effect of temperature, growth and reproduction on the estimated energy costs for each individual *N*. *cepedianus*, we then averaged energy expenditure across all individuals, to derive the mean routine energy expenditure of *N*. *cepedianus* in Norfolk Bay, assuming that our sample of 294 individuals is a reasonable representation of the population demographics at any given time. We then converted the units of routine energy expenditure from mg O_2_ h^−1^ to kJ sampling year^−1^, given there are 6048 h in a 36-week-period, and there is 68.3 mg O_2_ kJ^−1^ given a respiratory exchange ratio of 0.88^53^. We then used population estimates of *N*. *cepedianus* in Norfolk Bay at any given time^26^ to obtain the routine energy expenditure of the entire population while in Norfolk Bay from spring to autumn.

### Energy content of prey

The bioenergetics model constructed required an estimate of energy content for the key prey species of *N*. *cepedianus* in Norfolk Bay. Previous work identified the key prey species of *N*. *cepedianus* in Norfolk Bay^25^. The key prey species were categorized into fur seal *Arctocephalus pusillus*, other mammals, *M*. *antarcticus*, school shark *Galeorhinus galeus*, dogshark *Squalus acanthias*, unidentified sharks, eagle ray *Myliobatis tenuicaudatus*, Melbourne skate *Spiniraja whitleyi*, banded stingaree *Urolophus cruciatus*, unidentified batoids, elephantfish *Callorhinchus milii*, teleosts, and cephalopods (Table 1). The average available energy content (kJ) of each of these prey species or groups was calculated as the product of the energy-density of the tissue and their average body mass, multiplied by a factor of 0.73 to account for energy assimilation efficiency^54^. Tissue energy-density values were obtained for the various prey species or groups from bomb calorimetry measurements published in the literature, and where such data were unavailable we substituted for closely related species (Table 1). The body masses of the various chondrichthyan prey species were calculated by applying length-weight conversions derived using published and unpublished data (Table 1). The body lengths used in these length-weight conversions were recorded during long line sampling and gill-net surveys in Norfolk Bay and adjoining Frederick Henry Bay^24,55^; McAllister unpublished data; CSIRO, Australia, unpub. data. Average body mass for cephalopods was based on arrow squid *Nototodarus gouldi*, as it is abundant in the bay, and the most commonly consumed cephalopod species^25^. Average body mass of marine mammal was based on fur seal adult males and sub-adult of both sexes, which are the most common marine mammal in the Norfolk Bay region^56^. We assume that the whole-body of the prey is consumed by *N*. *cepedianus*, even if it is consumed by several individual *N*. *cepedianus*, as is likely the case for large prey items, such as mammals.

### Model simulations, variants and sensitivity analyses

To account for uncertainty in model structure and input parameters, we built variants of the bioenergetics model by incorporating parameter confidence through Monte Carlo simulations. Using our calculated value for the routine energy expenditure of the entire population of *N*. *cepedianus* in Norfolk Bay across the 36-week period each year, and the total energy available from each prey species (or group), we ran three model variants to estimate local predation rates by *N*. *cepedianus* on the various prey in Norfolk Bay. The three model variants provide estimates of *N*. *cepedianus* predation rates depending on the relative fraction that each prey species (or group) contributes to supporting the energy expenditure of *N*. *cepedianus*, which we based on three alternative techniques that are commonly used for measuring diet composition: (1) the frequency a prey species occurs in the diet, (2) the weight of each prey species (or group) as a fraction of the total weight of all prey consumed, and (3) the number of each prey consumed as a fraction of the total number of all prey consumed^33^.

The model estimates predation rate (P_*x*_; sampling year^−1^) on species *x* (or group *x*) over a sampling year following the equation, P_*x*_ = *MR* × F_*x*_/E_*x*_, where *MR* (kJ sampling year^−1^) is the routine energy expenditure of *N*. *cepedianus*, E_*x*_ is the available energy content (kJ) of species *x* (or group *x*), and F_*x*_ is the fraction of the diet of *N*. *cepedianus* represented by species *x* (or group *x*). Thus, the three model variants provide alternative estimates of F_*x*_, therefore leading to different estimates of P_*x*_. In model variant 1, which focuses only on the predation rate of *M*. *antarcticus*, F_*x*_ was set as 0.25 based on stomach flushing data that showed 25% of *N*. *cepedianus* sampled in Norfolk Bay had consumed *M*. *antarcticus* ^25^. In model variant 2, F_*x*_ is set as equal to the partly digested weight of each prey species (or group) in the stomach of *N*. *cepedianus*, divided by the total partly digested weight of all prey items present in the stomach. The weight of prey items was measured from regurgitated stomach contents at varying stages of digestion, obtained from stomach flushing *N*. *cepedianus* sampled in Norfolk Bay^25,45^. In model variant 3, F_*x*_ is the proportion that each prey contributes to the overall diet, calculated by multiplying the average weight of each prey (Table 1) by the number of that prey present in the stomach (Appendix S1), as determined from stomach flushing of *N*. *cepedianus* sampled in Norfolk Bay^25,45^. For the much larger mammalian prey species, ingestion weight was calculated as the average weight of the ingested pieces of mammal (Table 1). We only included pieces of mammal in the weight calculations that minimal digestion had occurred.

The three model variants were run using two alternative variations in the population size estimate of *N*. *cepedianus* in Norfolk Bay (Table 2). For the two population scenarios, we considered a log-normal distribution with mean of 562 ± 71 sharks, and another scenario with mean of 1130 ± 137 sharks^26^. These means are based on mark-recapture estimates spanning 20 and 44 weeks sampling, respectively. Given the potential temporal fluctuations in abundance in Norfolk Bay over the study period, both mean values are included in the model to span the potential range of abundance values for Norfolk Bay in this study. Natural fluctuations in abundance occur over days or weeks, as evident by tracking data that shows individual *N*. *cepedianus* move in and out of the bay during a season^26^. All simulations were done in the statistical package R^57^. For each of the six scenarios considered, 1000 Monte Carlo simulations were performed to account for uncertainty in *N*. *cepedianus* prey composition, body mass, and routine energy expenditure parameters (Q_10_, water temperature, the allometric power equation coefficient and the allometric power equation exponent) by drawing samples from a log-normal distribution with mean and standard deviation as presented in Table 1.

## Electronic supplementary material

Supplementary Information
