# Supplementary material for: The utility of bioenergetics modelling in quantifying predation rates of marine apex predators: Ecological and fisheries implications
Source: Sci Rep. 2017 Oct 11;7:12982. doi: 10.1038/s41598-017-13388-y (PMC5636836; doi:10.1038/s41598-017-13388-y)
Supplement: Supplementary file 1 — Supplementary Information [file 41598_2017_13388_MOESM1_ESM.pdf]

SUPPORTING INFORMATION

The utility of bioenergetics modelling in quantifying predation rates of marine apex predators: Ecological and fisheries implications

Barnett A, Braccini M, Dudgeon CL, Payne NL, Abrantes K, Sheaves M, Snelling EP

Appendix S1. %W corresponds to the percentage each prey contributes in weight of all prey consumed by *N. cepedianus* in Norfolk Bay, used in model variant 2 (Barnett et al. 2010b). Average %W was calculated based on the number of each prey consumed multiplied by its average weight (obtained from catch data), and the proportion each prey contributes to the overall weight of prey consumed, so that Average %W = (number × average weight for that species) × 100)/total average weight of all prey. This was used in model variant 3. Average weight was obtained for species caught in Norfolk Bay and surrounding bays (Table 1). Because entire seals/mammals were not consumed by an individual *N. cepedianus*, weight of undigested mammal occurring in stomach samples was used to obtain average weight of mammal consumed (Table 1).

**Species/group**	**%W**	**Average %W**
Fur seal *Arctocephalus pusillus*	35.3	7.2
Other mammal	7.1	3.4
Gummy shark *Mustelus antarcticus*	17.6	10.1
School shark *Galeorhinus galeus*	1.4	0.4
Dogshark *Squalus acanthias*	1.8	1.5
Unidentified shark	9.2	2.2
Eagle rays *Myliobatis tenuicaudatus*.	7.8	16.3
Melbourne skate *Spiniraja whitleyi*	9.5	42.5
Banded stingaree *Urolophus cruciatus*	1.7	0.4
Unidentified batiod	2.1	8.2
Elephantfish *Callorhynchus milii*	0.8	1.4
Teleosts (boney fish)	5.3	5.1
Cephalopods	0.3	1.3

Barnett, A., K. Abrantes, J. D. Stevens, J. L. Yick, S. D. Frusher, and J. M. Semmens. 2010b. Predator-prey relationships and foraging ecology of a marine apex predator with a wide temperate distribution. Marine Ecology-Progress Series **416**:189-200.

Appendix S2: Relative abundance of prey species in bays of Southern Tasmania. Taken from CSIRO gillnetting data from 1991 to 1997. CPUE is number of animals per gillnet set. See Stevens & West for fishing details. Gummy shark *M. antarcticus*, school shark *Galeorhinus galeus*, dogshark *Squalus acanthias*, eagle ray *Myliobatis tenuicaudatus*, Melbourne skate *Spiniraja whitleyi*, banded stingaree *Urolophus cruciatus*, elephantfish *Callorhinchus milii,* Port Jackson shark *Heterodontus portusjacksoni,* southern sawshark *Pristiophorus nudipinnis,* common sawshark *Pristiophorus cirratus*, piked spurdog *Squalus megalops,* draughtboard shark *Cephaloscyllium laticeps*, whitespotted skate *Dipterus cerva*, Australian angelshark *Squatina australis,* numb ray *Narcine tasmaniensis*, thornback skate *Dentiraja lamprieri*, sparsely-spotted stingaree *Urolophus paucimaculatus*, smooth stingray *Dasyatis brevicaudata*

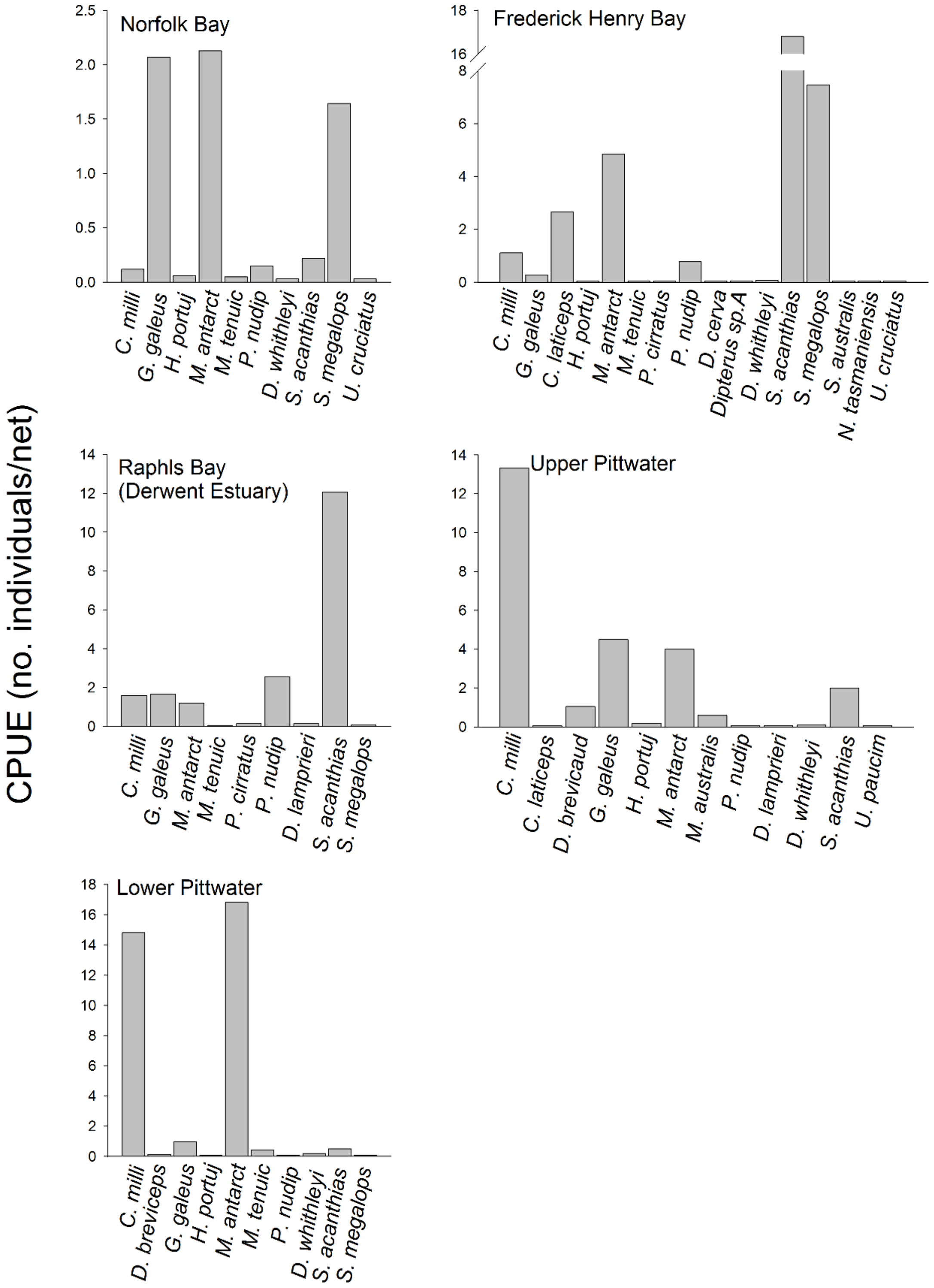
CPUE (no. individuals/net)
Norfolk Bay
C. milli
G. galeus
H. portuj
M. antarct
M. tenuic
P. nudip
D. whithleyi
S. acanthias
S. megalops
U. cruciatus
Frederick Henry Bay
C. milli
G. galeus
C. laticeps
H. portuj
M. antarct
M. tenuic
P. cirratus
P. nudip
D. cerva
Dipterus sp.A
D. whithleyi
S. acanthias
S. megalops
S. australis
N. tasmaniensis
U. cruciatus
Raphls Bay
(Derwent Estuary)
C. milli
G. galeus
M. antarct
M. tenuic
P. cirratus
P. nudip
D. lamprieri
S. acanthias
S. megalops
Upper Pittwater
C. milli
C. laticeps
D. brevicaud
G. galeus
H. portuj
M. antarct
M. australis
P. nudip
D. lamprieri
D. whithleyi
S. acanthias
U. paucim
Lower Pittwater
C. milli
D. breviceps
G. galeus
H. portuj
M. antarct
M. tenuic
P. nudip
D. whithleyi
S. acanthias
S. megalops
